# Decreased CD73+ Double-Negative T Cells and Elevated Level of Soluble CD73 Correlated With and Predicted Poor Immune Reconstitution in HIV-Infected Patients After Antiretroviral Therapy

**DOI:** 10.3389/fimmu.2022.869286

**Published:** 2022-04-04

**Authors:** Xinyue Wang, Leidan Zhang, Juan Du, Yuqing Wei, Di Wang, Chuan Song, Danying Chen, Bei Li, Meiqing Jiang, Mengyuan Zhang, Hongxin Zhao, Yaxian Kong

**Affiliations:** ^1^ Peking University Ditan Teaching Hospital, Beijing, China; ^2^ Beijing Key Laboratory of Emerging Infectious Diseases, Institute of Infectious Diseases, Beijing Ditan Hospital, Capital Medical University, Beijing, China; ^3^ Beijing Institute of Infectious Diseases, Beijing, China; ^4^ National Center for Infectious Diseases, Beijing Ditan Hospital, Capital Medical University, Beijing, China; ^5^ Clinical and Research Center of Infectious Diseases, Beijing Ditan Hospital, Capital Medical University, Beijing, China

**Keywords:** HIV, CD73, double-negative T cell, immune reconstitution, immune activation

## Abstract

Although extensive use of antiretroviral therapy (ART) has made great progress in controlling HIV replication and improving CD4^+^ T cell recovery, the immune reconstitution remained insufficient in some patients, who were defined as poor immunological responders (PIRs). These PIRs were at a high risk of AIDS-related and non-AIDS complications, resulting in higher morbidity and mortality rate. Thus, it is a major challenge and urgently needed to distinguish PIRs early and improve their immune function in time. Immune activation is a key factor that leads to impaired immune reconstitution in people living with HIV (PLWH) who are receiving effective ART. Double negative T cells (DNT) were reported to associate with the control of immune activation during HIV infection. However, the precise mechanisms by which DNT cells exerted their suppressive capacity during HIV infection remained puzzled. CD73, both a soluble and a membrane-bound form, display immunosuppressive effects through producing adenosine (ADO). Thus, whether DNT cells expressed CD73 and mediated immune suppression through CD73-ADO pathway needs to be investigated. Here, we found a significant downregulation of CD73 expression on DNT cells in treatment-naïve PLWH (TNs) compared to healthy controls, accompanied with increased concentration of sCD73 in plasma. Both the frequency of CD73^+^ DNT cells and the level of plasma sCD73 recovered after ART treatment. However, PIRs showed decreased percentage of CD73^+^ DNT cells compared to immunological responders (IRs). The frequency of CD73^+^ DNT cells was positively correlated with CD4^+^ T cell count and CD4/CD8 ratio, and negatively correlated with immune activation in PLWH. The level of sCD73 also showed a negative correlation to CD4^+^ T cell count and CD4/CD8 ratio. More importantly, in the present cohort, a higher level of sCD73 at the time of initiating ART could predict poor immune reconstitution in PLWH after long-term ART. Our findings highlighted the importance of CD73^+^ DNT cells and sCD73 in the disease progression and immune reconstitution of PLWH, and provided evidences for sCD73 as a potential biomarker of predicting immune recovery.

## Introduction

In recent decades, extensive use of antiretroviral therapy (ART) in people living with HIV (PLWH) has made continued progress in controlling viral replication and restoring CD4^+^ T cell count (1). However, part of patients (from 15% to 30%) remained insufficient in immune recovery, leading to a high risk of AIDS-related and non-AIDS complications. These patients were classified as poor immunological responders (PIRs), which had higher rates of morbidity and mortality than immunological responders (IRs) (2–5). Thus, it is a major clinical challenge and urgently needed to identify PIRs early and improve their immune homeostasis in time.

Persistent immune activation played a prominent role in the loss of CD4^+^ T cells and disease progression during HIV infection (6, 7). High levels of immune activation occurred early in primary HIV infection and still existed in PLWH who were receiving effective ART (8, 9). Indeed, immune activation is a key factor that leads to impaired immune reconstitution (10, 11). The successful control of immune activation could be a target in clinical therapy for improving damaged immune recovery. Double negative T cells (DNT cells) were characterized to express CD3 and TCRαβ, but not NK cell markers (12, 13). Although DNT cells comprised only 1–3% of total T cells in peripheral blood and lymphoid organs of humans and mice, these cells were crucial for maintaining immune homeostasis (14–17). A series of previous studies indicated that DNT cells were predominately memory phenotype in SIV infection, and have a T helper function, secreting CD4-like cytokines (IL-4, IL-17, IFN-γ and TNF-α) in SIV-infected or healthy monkeys such as sooty mangabeys (18–20). However, most studies identified these cells as similar to regulatory T cells (Tregs), which could suppress T cells, B cells, dendritic cells, and NK cells in graft-versus-host disease, autoimmune diseases, and infectious disease (21–23). Elevated DNT cells were reported to associate with the control of immune activation in both primary HIV-1 infection and IRs, suggesting the role of DNT cells in disease progression and immune reconstitution of PLWH (23, 24).

However, the precise mechanisms by which DNT cells exert their suppressive capacity during HIV infection remained puzzled. DNT cells with distinct phenotype and function can mediate inhibition *via* different mechanisms, including secretion of TGF-β, IL-10, perforin, and interaction of CTLA-4, which also involved in Treg-mediated immune suppression (25–28). Nevertheless, extracellular adenosine (ADO), which participated in the suppressive activity of Tregs, has never been noticed in DN T cells (29). ADO is a critical regulator of innate and adaptive immune responses, inhibiting T cell proliferation and the secretion of inflammatory cytokines, including IL-2, TNFα, and IFN-γ (30, 31). The ADO production is generally regulated by two enzymes sequentially, CD39 and CD73, which were expressed by many different cell types including immune cells (32–34). CD39 firstly catalyzes the dephosphorylation of circulating ATP and ADP to 5’-AMP. After that, CD73, an ecto-5’-nucleotidase existing in a soluble or membrane-bound form, converts AMP to ADO (35, 36). Several studies have confirmed the absence of CD73 on Tregs in human despite the key role of CD39 and ADO in immune suppression of Tregs (35). Thus, whether DNT expressed CD73 and mediated immune suppression through CD73- ADO pathway needs to be investigated.

In the present study, we found that CD73^+^ DNT cells in PLWH were associated with the control of immune activation, disease progression and immune reconstitution after ART. Of interest, soluble CD73 (sCD73) was also involved in immune suppression during HIV infection. Importantly, we provided evidence that elevated baseline levels of sCD73 could predict poor immune recovery in PLWH after long-term ART.

## Materials and Methods

### Study Participants

The study was approved by the Committee of Ethics at Beijing Ditan Hospital, Capital Medical University in Beijing with informed consent acquired from all participants. We conducted a cross-sectional study in 263 PLWH and 25 gender and age-matched healthy control subjects (HCs). These PLWH included 193 treatment-naïve patients (TNs), 49 IRs, and 21 PIRs. IRs were PLWH with CD4^+^ T cell count≥350 cells/μl who had experienced ART for 5.9 years (IQR 5.1-7.4) with undetectable viral load (<50 copies/ml) according to routine clinical assays. PIRs were PLWH with CD4^+^ T cell count<350 cells/μl who had experienced ART for 5.5 years (IQR 5.0-6.2) with undetectable viral load. The demographic and clinical characteristics of the participants were shown in **Table 1**.

**Table 1:** Demographic and clinical characteristics of study participants.

Characteristics	HCs	TNs			IRs	PIRs
		CD4≥350	200≤CD4<350	CD4<200		
N (%)	25	66 (34.20)	71 (36.79)	56 (29.01)	49	21
Sex (M/F)	24/1	64/2	68/3	56/0	48/1	21/0
Age (mean, years)	34 ± 7	32 ± 10	31 ± 7	35 ± 8	36 ± 6	39 ± 7
CD4 count (cells/mm^3^), median (IQR)	–	456 (408-529)	281 (244-312)	83 (33-146)	628 (560-743)	271 (229-328)
CD8 count (cells/mm^3^), median (IQR)	–	1085 (796-1401)	1017 (727-1267)	729 (547-904)	788 (656-1009)	691 (600-790)
CD4/CD8 ratio, median (IQR)	–	0.43 (0.31-0.61)	0.28 (0.22-0.37)	0.11 (0.05-0.17)	0.77 (0.64-1.06)	0.43 (0.36-0.49)
HIV RNA viral load (copies/mL), median (IQR)	–	11091 (4239-29660)	35908 (10658-119660)	82353 (26229-211286)	<LDL	<LDL

HC, healthy controls; TN, treatment-naive HIV-1-infected patients; IR, immunological responders; PIR, poor immunological responders; M, male; F, female; LDL, lower detection limit. TNs are divided into three subgroups according to blood CD4^+^ T cell count.

In the retrospective cohort study, we recruited 171 participants, who were PLWH with baseline CD4^+^ T cell count<350 cells/μl and had received ART for 4.9 years (IQR 4.5-5.2) with undetectable viral load according to routine clinical assays. These participants were divided into IRs (CD4^+^ T cell count≥350 cells/μl) and PIRs (CD4^+^ T cell count<350 cells/μl) according to their CD4^+^ T cell count at 4.9 years following ART initiation. The demographic and clinical characteristics of the participants were described in **Table S1**.

### Separation of Peripheral Blood Mononuclear Cells (PBMCs)

PBMCs were collected from the peripheral blood in EDTA-K_2_ tubes using standard Ficoll-Paque gradient centrifugation. All samples were processed and analyzed within 24 hours of collection.

### Plasma HIV-1 Viral Load and CD4^+^ T-Cell Count

The HIV-1-RNA levels in plasma were quantified using a Standard Amplicor HIV Monitor assay, version 1.5 (Roche Diagnostics, Indianapolis, IN, USA), with a limit of detection of 40 copies/ml. The CD4^+^ T-cell count was measured by a standard flow cytometry technique with a TruCOUNT tube in routinely equipped laboratories (BD Biosciences, San Jose, CA, USA).

### Immunofluorescence Staining and Flow Cytometry Analysis

The expression of ectonucleotidases (CD39 and CD73) on αβDNT and activated Treg cells was evaluated by flow cytometry. The gating strategy for activated Treg cells was performed as previously described (5). PBMCs were stained with directly conjugated antibodies for 30 min at 4°C in the dark. The cells were washed before flow cytometry analysis. Antibodies used included anti-human CD3-BV785 (clone SK7), CD4-APC-fire750 (clone SK3), αβTCR-BV421 (clone IP26), CD56-BV510 (clone HCD56), CCR7 (CD197)-PE-cy7 (clone G043H7), HLA-DR-AF700 (clone L243), CD73-PE (clone AD2), CD39-BV605 (clone A1, BioLegend, San Diego, CA, USA), CD45RA-BV711 (clone HI100), CD38-BUV737 (clone HB7), CD25-PE-CF594 (clone M-A251), CD8-FITC (clone SK1), CD8-BUV395 (clone RPA-T8, BD Biosciences, San Diego, CA, USA), and the corresponding isotype controls. Data were acquired on a BD LSR Fortessa flow cytometer (BD Biosciences) and analyzed with FlowJo software (Tree Star, Ashland, OR, USA).

### Quantification of Soluble Markers

Plasma samples from healthy controls and patients were assayed for the levels of different cytokines and chemokines (IFN-γ, IFN-α, IL-18, IL-1β, IL-15, TNF-α, IL-2, RANTES, MIP-1β, IP-10, and IL-7) using Luminex multiple kits (Invitrogen, Carlsbad, CA, USA). Plasma levels of sCD73 were analyzed by ELISA (Abcam, ab213761) according to the manufacturer’s protocol. Adenosine was detected in plasma by Adenosine Assay Kit (Fluorometric) (Abcam, ab211094).

### Statistical Analysis

The data are expressed as the mean (standard deviation, SD), median (interquartile range, IQR), and percentage. SPSS21 (IBM Corporation, New York, NY, USA) and GraphPad8 (GraphPad Software, La Jolla, CA, USA) were used for statistical calculations. The normality of each variable was evaluated using the Kolmogorov-Smirnov test. For normally distributed data, the comparison of two variables was performed using unpaired two-tailed Student’s t-tests. When the data were not normally distributed, the comparison of variables was performed with a Mann-Whitney *U* test or a Wilcoxon matched-pairs signed-rank test for unpaired and paired data, respectively. In the case of comparing two more independent samples, the Kruskal-Wallis test followed by Dunn’s multiple comparisons test was applied. Correlation coefficients were calculated for nonparametric distributions using Spearman’s correlation test. The Chi-square test was used to compare categorical variables.

When performing univariable and multivariable Cox regression analyses, continuous variables of soluble factors were converted into categorical variables according to cutoff value (with the highest Youden index) which was obtained by the receiver operating characteristic (ROC) curve. Significant factors (P<0.05) from univariable Cox proportional regression analysis were included in the multivariable analysis to identify factors independently associated with immune reconstitution expressed as hazard ratios and related 95% confidence intervals (95%CI). Survival analysis was performed using a Kaplan–Meier survival plot, and the log-rank test P value was calculated. The endpoint of Kaplan-Meier and Cox regression analyses was CD4^+^ T cell recovery or restoration of CD4/CD8 ratio, which was defined by the data of the first values of CD4^+^ T cell count≥350 cells/μl or CD4/CD8 ratio≥0.7. Receiver operating characteristic (ROC) was used to evaluate the predicted potential (with 95% confidence intervals), and to calculate the sensitivity and specificity. P<0.05 was considered statistically significant.

## Results

### Decreased Frequency of CD73^+^ DNT Cells in PLWH

**Figure 1:**
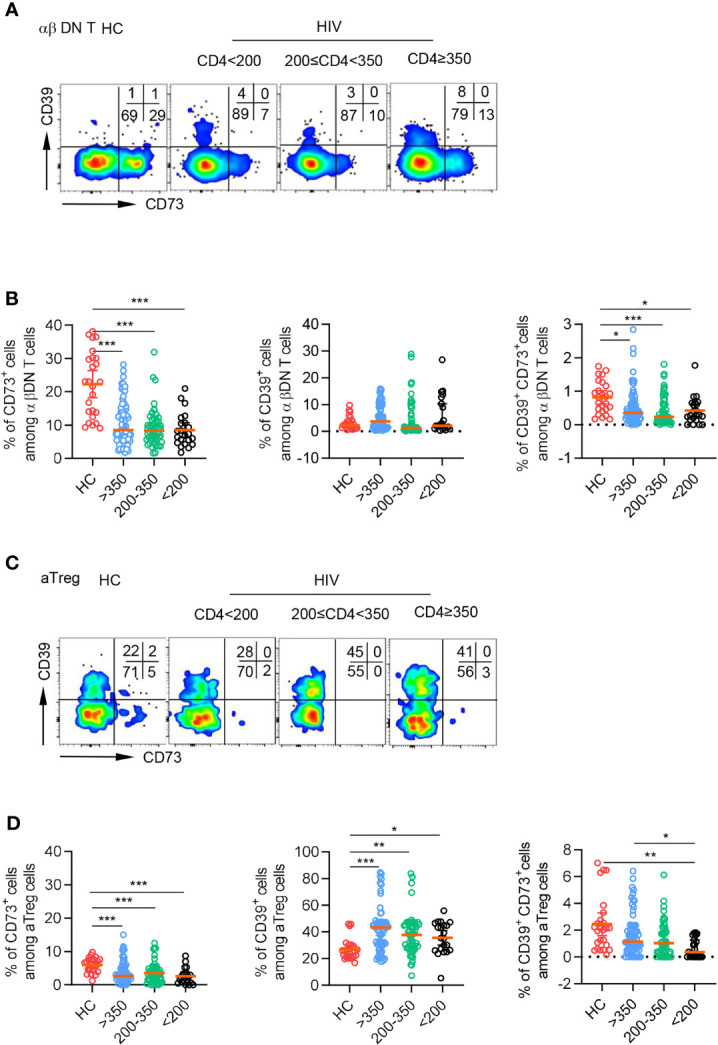
CD73 was downregulated on DNT cells from TNs compared to HCs. Flow cytometry analysis of CD39 and CD73 expression was performed on PBMCs collected from HCs and different TNs groups. **(A)** Representative flow data showed the expression of CD39 and CD73 gated on DN T cells from HCs and different TNs groups. **(B)** Scatter plots of the percentage of CD73^+^, CD39^+^ and CD39^+^ CD73^+^ DN T cells from HCs and different TNs groups (n=22-66 each group). *P* values were obtained by Kruskal-Wallis test followed by Dunn’s multiple comparisons test. **(C)** Representative flow data showed the expression of CD39 and CD73 gated on activated Treg cells from HCs and different TNs groups. **(D)** Scatter plots of the percentage of CD73^+^, CD39^+^and CD39^+^ CD73^+^ activated Treg cells from HCs and different TNs groups (n=21-66 each group). *P* values were obtained by Kruskal-Wallis test followed by Dunn’s multiple comparisons test. *P<0.05, **P<0.01, ***P<0.001.

To investigate the potential role of CD39 and CD73 on DNT cells in HIV infection, we recruited 193 TNs and 25 gender and age-matched HCs. TNs were subdivided into three groups according to their CD4^+^T cell count (<200 cells/µl, 200-350 cells/µl, and≥350 cells/µl). The demographic and clinical characteristics of these patients were shown in **Table 1**. We examined the expression pattern of CD73 and CD39 on DNT cells by flow cytometry. Representative flow cytometry gating strategy for αβDNT cells was shown in **Figure S1**. DNT cells preferentially expressed CD73 in HCs and TNs compared with CD39 (**Figure 1A**). Despite a significant increase in the absolute number and percentage of DNT cells from TNs (**Figures S2A, B**), the patients showed significantly decreased level of CD73 and comparable expression of CD39 on DNT cells compared to HCs. Accordingly, there was a lower frequency of CD73^+^CD39^+^ DNT cells in TNs than in HCs (**Figure 1B**). As previously reported, we also found that activated Tregs (aTregs) expressed high levels of CD39 and almost did not express CD73 in both HCs and TNs (**Figures 1C, D**), implying CD73^+^ DNT cells as an assistant of aTregs in producing ADO. In all, these data suggested the involvement of CD73^+^ DNT cells during HIV infection.

### Decreased Frequency of CD73^+^ DNT Cells Was Partly Reversible After ART and Associated With CD4^+^ T Cell Count and Immune Activation in PLWH

**Figure 2:**
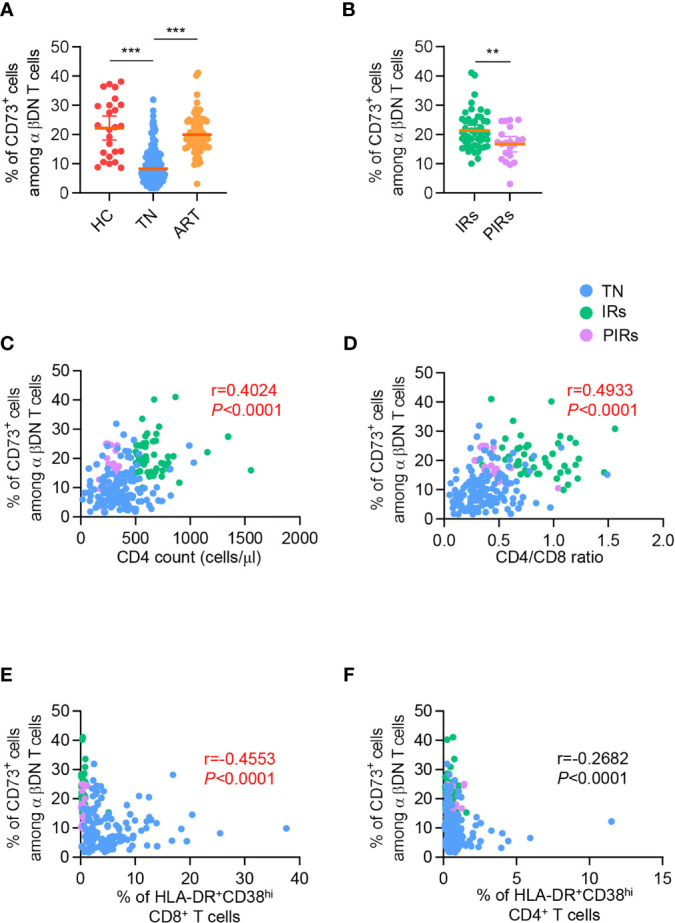
The frequency of CD73^+^ DN T cells was partly recovered after ART and correlated with CD4 count, CD4/CD8 ratio, and immune activation in PLWH. **(A)** Scatter plots displayed the frequency of CD73^+^ DN T cells from HCs, TNs, and ART-experienced PLWH (n=25-139 each group). *P* values were obtained by the Kruskal-Wallis test followed by Dunn’s multiple comparisons test. **(B)** Comparison of the frequency of CD73^+^ DN T cells between IRs and PIRs with matched baseline CD4 count (n=21-49 each group). *P* values were obtained by unpaired t-test. **(C–F)** Correlations between percentage of CD73^+^ DN T cells with CD4 count **(C)**, CD4/CD8 ratio **(D)**, HLA-DR^+^CD38^hi^ CD8^+^ T cells **(E)**, HLA-DR^+^CD38^hi^ CD4^+^ T cells **(F)**. Spearman’s non-parametric test was used to test for correlations. **P<0.01, ***P<0.001.

To further investigate the role of CD73^+^ DNT cells in immune reconstitution after ART, we then assessed the expression of CD73 on DNT cells among patients experiencing 5.7 years of ART. As shown in **Figure 2A**, PLWH who experienced long-term ART displayed elevated frequency of CD73^+^ DNT cells compared to TNs. Meanwhile, the percentage of DNT cells in PLWH with ART was lower than in TNs (**Figures S2C**). We further divided 70 ART-treated PLWH into two subgroups: 49 IRs with CD4^+^ T cell count≥350 cells/µl and 21 PIRs with CD4^+^ T cell count<350 cells/µl after 5.7 years of ART. The percentage of CD73^+^ DNT cells was significantly higher in IRs than PIRs, despite comparable frequencies of DNT cells between these two groups, implying that the low frequency of CD73^+^ DNT cells was associated with incomplete restoration of CD4^+^ T cells (**Figure 2B** and **Figure S2D**).

We next applied correlation analysis in all PLWH, including TNs and ART-treated patients. It was revealed that the frequency of CD73^+^ DNT cells was positively correlated with CD4^+^ T cell count (r=0.4024, *P*<0.0001; **Figure 2C**) and CD4/CD8 ratio (r=0.4933, *P*<0.0001; **Figure 2D**), whereas no significant correlation was observed between the percentage of CD73^+^ DNT cells and virus load (**Figure S3A**). Of note, CD73^+^ DNT cells exhibited a significant negative correlation with the percentage of HLA-DR^+^CD38^hi^ CD8^+^ T cells instead of the CD4^+^ T fraction (r=-0.4553, *P*<0.0001; r=0.2682, *P*<0.0001, respectively; **Figures 2E, F**). In addition, we also assessed the correlation of DNT cell frequency with CD4^+^ T cell count, CD4/CD8 ratio, and immune activation and found no correlation among these parameters (**Figures S2E**–**H**). Collectively, these results suggested that the depletion of CD73^+^ DNT cells was associated with uncontrolled immune activation and adverse clinical outcomes in PLWH.

### Increased Level of sCD73 Was Related to Disease Progression and Clinical Outcomes in PLWH

**Figure 3:**
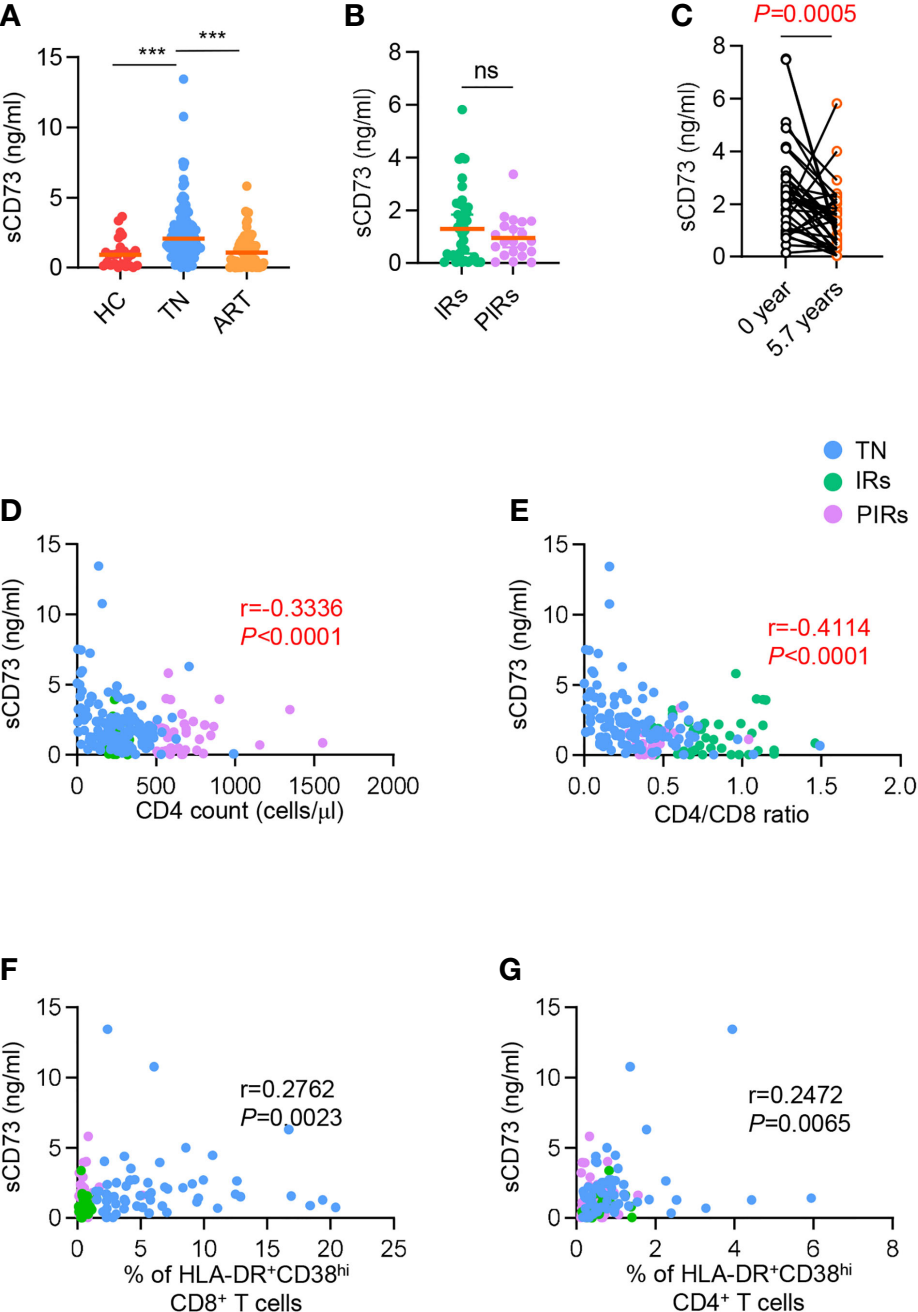
Elevated sCD73 was reversed after ART and associated with clinical outcome. **(A)** Data were shown as scatter plots comparing the concentration of sCD73 in plasma among HCs, TNs, and ART-experienced PLWH (n=25-110 each group). *P* values were obtained by Kruskal-Wallis test followed by Dunn’s multiple comparisons test. **(B)** Scatter plots depicting the concentration of sCD73 among IRs and PIRs with matched baseline CD4 count (n=21-41 each group). *P* values were obtained by Mann-Whitney test. **(C)** Longitudinal analysis of sCD73 at baseline and 5.7 years or above following ART (n=38). *P* values were obtained by Wilcoxon matched-pairs signed-rank test. **(D-G)** Correlation analysis of sCD73 level with CD4 count **(D)**, CD4/CD8 ratio **(E)**, HLA-DR^+^CD38^hi^ CD8^+^ T cells **(F)**, and HLA-DR^+^CD38^hi^ CD4^+^ T cells **(G)**. Spearman’s non-parametric test was used to test for correlations. ***P<0.001. ns, not significant.

Considering the low expression of CD73 on DNT cells in TNs, this raises an important question: whether the concentration of sCD73 in plasma has also changed. First, we observed a significant increase of sCD73 in plasma from TNs compared to HCs, whereas the level of sCD73 was downregulated in ART-treated patients (**Figure 3A**). However, there were comparable levels of sCD73 in IRs and PIRs (**Figure 3B**). We next analyzed the longitudinal data of sCD73 from PLWH at the baseline and 5.7 years after ART initiation. The concentration of sCD73 was obviously downregulated after 5.7 years ART (**Figure 3C**). Considering that the membrane-bound form of CD73 expressed by DNT cells was involved in the control of immune activation and CD4^+^ T cell recovery in PLWH, we speculated that sCD73 might play a role in HIV infection. Consistently, the level of sCD73 was inversely correlated with CD4^+^ T cell count and CD4/CD8 ratio, respectively (r=-0.3336, *P*<0.0001; r=-0.4114, *P*<0.0001; **Figures 3D, E**). However, no correlation was found between sCD73 and the percentage of HLA-DR^+^CD38^hi^ cells among CD8^+^ and CD4^+^ T cells (r=0.2762, *P*=0.0023, r=0.2472, *P*=0.0065, respectively; **Figures 3F, G**). Accordingly, there was no correlation between sCD73 and virus load (**Figure S3B**).

Since CD8 T cells were reported to contribute to ADO-mediated immune suppression by releasing CD73-containing extracellular vesicles, we tested the correlation of sCD73 or ADO with CD73^+^ CD8 T cells and CD73^+^ DNT cells, and correlation between ADO and sCD73. Of interest, the concentration of sCD73 was inversely related to the frequency of CD73^+^ CD8 T cells but not CD73^+^ DNT cells in TNs (r=-0.3011, *P*=0.0216; r=-0.04427, *P*=0.7414, respectively; **Figures S4A, B**). Oppositely, in PLWH who experienced ART, the level of sCD73 showed a negative correlation with CD73^+^ DNT cells but not CD8 subset (r=-0.3220, *P*=0.0107; r=-0.1289, *P*=0.3184, respectively; **Figures S4C, D**). No correlation was observed between ADO and CD73^+^ DNT, CD73^+^ CD8^+^ T cells, and sCD73 (**Figure S5**). These data could provide an explanation for the derivation of sCD73 in different immune status of PLWH.

### Plasma sCD73 Was Identified as a Predictive Factor for Immune Reconstitution in PLWH After Long-Term ART

To further identify the potential predictive role of baseline sCD73 in clinical outcomes of ART-treated PLWH, we performed a retrospective cohort study of 171 patients who had a baseline CD4^+^ T cell count lower than 350 cells/μl and experienced ART for 4.9 years with good viral responses. The detailed demographic and clinical information were shown in **Table S1**. Based on the baseline level of sCD73 at the time of initiating ART, these 171 patients were classified into high-sCD73 (sCD73>4.111 ng/ml) vs. low-sCD73 (sCD73 ≤ 4.111 ng/ml) subgroups. We analyzed the incidence of poor immune reconstitution in these two groups after different duration of ART. It was revealed that patients with high sCD73 exhibited a greater risk of poor immune reconstitution after 1 to 5 years of ART than those with low sCD73 (**Table S2**).

**Figure 4:**
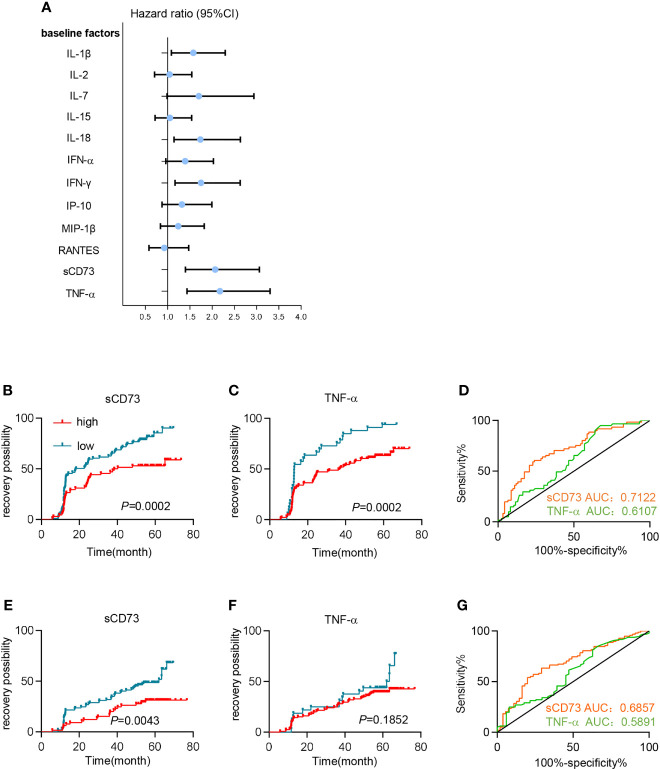
High levels of sCD73 predicted poor immune reconstitution in PLWH. **(A–D)** Patients’ clinical outcomes were defined by CD4 cell count. **(A)** Univariate Cox regression analyzed the association between different plasma soluble factors at baseline and immune recovery. Dot and error bars represent the regression coefficients with 95% CI. **(B, C)** Kaplan-Meier curves estimate recovery possibility for patients in high-concentration (red line) and low-concentration groups (blue line) segregated by **(B)** sCD73 (n=68 [high], n=99 [low]) and **(C)** TNF-α (n=129 [high], n=33 [low]). *P* values were obtained by Log-rank test. **(D)** Receiver operating characteristic (ROC) analyses for sCD73 (n=153, yellow line) and TNF-α (n=147, green line). **(E–G)** Patients’ clinical outcomes were defined by CD4/CD8 ratio restoration. **(E, F)** Kaplan-Meier curves estimate recovery possibility for patients in high-concentration (red line) and low-concentration groups (blue line) segregated by **(E)** sCD73 (n=65[high], n=97[low]) and **(F)** TNF-α (n=125[high], n=32[low]). *P* values were obtained by Log-rank test. **(G)** Receiver operating characteristic (ROC) analyses for sCD73 (n=153, yellow line) and TNF-α (n=147, green line).

In addition to sCD73, we further detected the baseline concentrations of various cytokines and chemokines, which were reported to associate with disease progression or clinical outcomes during HIV infection, including IFN-γ, IFN-α, IL-18, IL-1β, IL-15, TNF-α, IL-2, RANTES, MIP-1β, IP-10, and IL-7. As shown in **Figure 4A** and **Table S3**, we performed a univariate analysis and found that increased baseline levels of IL-1β (*P*=0.017), IL-18 (*P*=0.009), IFN-γ (*P*=0.007), TNF-α (*P*<0.0001), and sCD73 (*P*<0.0001) were significantly associated with a higher risk of poor immune reconstitution in PLWH. We next included these factors in a multivariate Cox regression analysis, and showed that sCD73 (HR 2.057 [95%CI 1.356-3.121]; *P*=0.001) and TNF-α (HR 1.913 [95%CI 1.241-2.950]; *P*=0.003) both remained as independent factors affecting CD4^+^ T cell recovery for ART-treated PLWH.

Furthermore, we sought to utilize sCD73 and TNF-α to predict clinical outcomes. When using optimal cutoff values, patients with high baseline levels of sCD73 or TNF-α demonstrated an elevated incidence of PIRs based on Kaplan-Meier (K-M) curves (*P*=0.0002, and *P*=0.0002, respectively; **Figures 4B, C**). More importantly, Receiver operating characteristic (ROC) analysis showed that sCD73 had a higher area under the curve (AUC) (0.7122 [95%CI 0.6291-0.7953]) than TNF-α (AUC 0.6107 [95%CI 0.5202-0.7012]; **Figure 4D**). Additionally, we also defined the clinical outcomes according to CD4/CD8 ratio restoration. Patients were classified into two subgroups: patients with high ratio restoration who achieved CD4/CD8 ratio of 0.7 or above after 4.9 years ART, and patients with low ratio restoration who failed to achieve CD4/8 ratio of 0.7 after 4.9 years ART. Similar results were shown in sCD73 (**Figure 4E**). However, TNF-α may not be a potential predictor for the CD4/CD8 ratio restoration (**Figures 4F, G**).

In summary, sCD73 in plasma represented a potential predictive biomarker of immune reconstitution after ART.

## Discussion

Previous studies have identified the crucial role of CD39/ADO pathway in immune suppression of Tregs during chronic HIV infection (37–39). Due to the lack of CD73 in Tregs, the source of CD73 and its essential role in AIDS progression remained puzzled and needed to be well investigated. In the present study, we demonstrated the involvement of CD73^+^ DNT cells in chronic HIV infection and disease progression. We found that the frequency of CD73^+^ DNT cells in PLWH, including TNs and those who experienced long-term ART was associated with CD4^+^ T cell count and immune activation. Additionally, the serum concentration of sCD73 showed a negative correlation with CD73^+^ DNT cells in PLWH who experienced ART. More importantly, the level of sCD73 at the baseline could predict CD4^+^ T cell recovery in PLWH. Patients with higher baseline levels of sCD73 had a higher risk of poor immune reconstitution. To our knowledge, this is the first evidence for a predictive role of sCD73 in immune reconstitution after long-term ART.

It is acknowledged that a significant percentage of ART-treated patients, classified as PIRs, failed to achieve optimal CD4^+^ T cell recovery, despite the HIV RNA being undetectable after several years of ART (4). These PIRs had a higher risk of AIDS-related and non-AIDS complications, such as cardiovascular disease, bone, and renal disease, neurocognitive decline, and premature aging (40–43). Notably, PIRs had higher rates of morbidity and mortality than IRs (5). Thus, there is an urgent need to identify PIRs early and provide effective treatment as soon as possible. Most prior studies indicated that the potential baseline predictors of poor immune reconstitution contained several clinical features (such as age, co-infection, CD4^+^ T cell count, CD8 count, CD4/CD8 ratio, and viral load), immune activation (CD38^+^HLA-DR^+^), genetic factors (CCR5 polymorphisms, IL7RA polymorphisms and mitochondrial haplogroups), and thymic function (44–52). In fact, the baseline CD4^+^ T cell count is the most recognized method for predicting the immune recovery after ART, however, it remains a considerable challenge to acquire this data in resource-limiting settings. Additionally, in contrast to stable serological biomarkers, CD4^+^ T cell count is susceptible to several factors, including physiology, emotion, drugs, age, and even the skill of lab technicians. Indeed, a series of studies indicated that several plasma biomarkers of systemic inflammation after ART initiation are potentially more predictive for future non-AIDS complications or CD4^+^ T cell recovery than cellular markers of immune activation (53–55).

Here, we took sCD73 and eleven other cytokines, which were indicated to associate with disease progression or clinical outcomes in PLWH, into the present study (56–64). Based on a series of rigorous statistical analysis, only the baseline concentration of sCD73 was identified as the most important predictive biomarker for immune reconstitution after ART. However, Prebensen et al. pointed out that elevated baseline levels of MIP-1β identified long-term PIRs who started ART at CD4^+^ T cell counts<200 cells/μl, which was not consistent with our study (64). Several possibilities were considered to explain the contradiction. On one hand, our cohort included PLWH with a baseline CD4^+^ T cell count<350 cells/μl significantly higher than their baseline CD4^+^ T cell count. Distinct immune characteristics and responses in these two cohorts could contribute to the heterogeneity of the results. On the other hand, they did not perform accurate predictive analysis, but only binary logistic regression, which was not enough to conclude a predictive biomarker.

More importantly, we evaluated the clinical outcomes not only by CD4^+^ T cell count but also by using CD4/CD8 ratio, which has been increasingly recognized as a biomarker of immune reconstitution during HIV treatment (65, 66). Indeed, CD4/CD8 ratio has been recommended as a stronger predictor of poor outcomes than the CD4^+^ T cell count in some clinical guidelines (67). It was gradually recognized that CD4^+^ T cell count does not reflect immune activation and risk of non-AIDS-defined events, despite its crucial role in monitoring immune recovery (65). Instead, CD4/CD8 ratio was found to independently associate with immune activation and serious non-AIDS events (68–71). In fact, severe immune defects were also identified in some PLWH with virological suppression who had CD4^+^ T cell count of more than 500 cells/μl but a low CD4/CD8 ratio (65, 72). Therefore, immune reconstitution and prognosis after ART were well monitored by both CD4^+^ T cell count and CD4/CD8 ratio. In the present study, we found that sCD73 instead of TNF-α could predict CD4^+^ T cell recovery as well as the restoration of CD4/CD8 ratio, further demonstrating a predictive value of sCD73 for the clinical outcome.

Of surprise, we observed reduced CD73^+^ DNT cells accompanied with an accumulation of CD39^+^ Tregs in TNs, despite CD73 being a similar rate-limited enzyme as CD39 in CD39/CD73/ADO pathway (30). In addition, CD73 was also down-regulated in CD8 T cells and B cells during HIV infection (73–75). The distinct dynamics of CD73 and CD39 during HIV infection might be explained by the increased plasma level of sCD73 in PLWH. The notion was further supported by a higher enzymatic activity of CD73 in its soluble form than the membrane-bound variant (76, 77). Thus, we speculated that CD73^+^ immune cells might release sCD73 in an inflammatory environment, which could cooperate with CD39^+^ Treg to produce extracellular ADO for immune suppression. Interestingly, the concentration of sCD73 was related to the frequencies of CD73^+^ CD8 T cells in TNs, while in ART-treated patients, the sCD73 level correlated with CD73^+^ DNT cells. It was implied that the major source of sCD73 might come from different cells in different immune status, which needed to be further explored. Considering that CD73 was widely expressed on various types of immune cells, further investigation was urgently needed to determine the exact origin of sCD73 in the future.

However, the exact mechanisms of increased sCD73 in plasma during HIV infection still remained in puzzle. To our knowledge, soluble form of CD73 can be cleaved from the cellular membrane or secreted by various tumor cells (78–81). Previous studies showed that the decrease of the membrane-bound CD73 activity on activated CD8^+^ T cells was accompanied by an increase in the concentration of soluble counterpart, and paralleled by elevated generation of ADO in the cell culture supernatant of activated cells. Similar results were observed on CD8^+^ T cells when using phosphatidylinositol-specific phospholipase C (PI-PLC) to force shedding of cell surface CD73 (76–78). Thus, considering the inverse trend between cell-bound and soluble forms of CD73 in our study, we preferred to assume that sCD73 were produced by cleavage of membrane-bound counterpart, although the source of secretion could not be completely ruled out.

Given that ADO was a key factor in CD73-mediated immunosuppression, we also detected the concentration of ADO in plasma from PLWH. Unfortunately, no correlation was observed between ADO and sCD73, CD73^+^ DNT, and CD73^+^ CD8^+^ T cells, which could be explained by several possibilities. First, ADO was mainly generated in the local environment of tissue injury, hypoxia, or inflammation where high local concentrations of ATP and ADP would be released and correspondingly exerted its immune effects restricted locally. Thus, the plasma concentration of ADO was hard to reflect their concentration and activity in the local environment owing to dilution and time of circulation (82–84). Second, due to the rapid formation and rapid clearance of ADO in blood, it was challenging to determine accurate concentrations of extracellular ADO in blood samples (84). Finally, the purinergic signaling is a complex network including a number of purinergic ligands, receptors, enzymes, channels, and transporters. In addition to CD73, many other purine-metabolizing enzymes, including adenosine deaminase (ADA), CD38, ATP-degrading enzymes like ENPPs and CD39, as well as ATP-regenerating kinases were also present in human plasma (78, 84–86). Thus, the plasma concentration of ADO could be mediated by multiple pathways, implying a complicated relationship of ADO with CD73-expressing cells or sCD73.

The present study has several limitations. First, due to a minimal number of DNT cells in PLWH, it is difficult to perform *in vitro* functional experiments to verify the role of CD73 in the inhibition mediated by DNT cells and the origin of sCD73. Second, we did not take the frequency of CD73^+^ DNT cells into the predictive model for clinical outcomes of PLWH, owing to the lack of the baseline flow cytometry data of the retrospective cohort. Third, the confounding factor of therapy initiation was not excluded from the correlation analysis in the present study. Finally, only a single-center cohort study was conducted for the predictive analysis; we need to recruit more cohorts in additional sites to verify our findings.

In summary, our study demonstrated that a higher concentration of sCD73 at the baseline could predict poor CD4^+^ T cells recovery in PLWH after long-term ART, providing a potential biomarker for detecting immune reconstitution early.

## Data Availability Statement

The original contributions presented in the study are included in the article/**Supplementary Material**. Further inquiries can be directed to the corresponding authors.

## Ethics Statement

The studies involving human participants were reviewed and approved by the Committee of Ethics at Beijing Ditan Hospital. The patients/participants provided their written informed consent to participate in this study.

## Author Contributions

XW, LZ, and JD performed the experiments and analyzed the data. YW, DW, CS, DC, BL, MJ, and MZ collected samples, and performed the experiments. HZ participated in the critical review of the manuscript and revised the manuscript. YK designed the experiments, analyzed the data and wrote the manuscript. All authors contributed to the article and approved the submitted version.

## Funding

This work was supported by National Natural Science Foundation of China (82171548, 81971307), Beijing Municipal Natural Science Foundation for Distinguished Young Scholars (JQ21023), Beijing Municipal Administration of Hospitals’ Ascent Plan (DFL20191802), and Beijing Municipal Administration of Hospitals Clinical Medicine Development of Special Funding Support (ZYLX202126).

## Conflict of Interest

The authors declare that the research was conducted in the absence of any commercial or financial relationships that could be construed as a potential conflict of interest.

## Publisher’s Note

All claims expressed in this article are solely those of the authors and do not necessarily represent those of their affiliated organizations, or those of the publisher, the editors and the reviewers. Any product that may be evaluated in this article, or claim that may be made by its manufacturer, is not guaranteed or endorsed by the publisher.

## References

[1] Zhang LX Jiao YM Zhang C Song JW Fan X Xu RN. HIV Reservoir Decay and CD4 Recovery Associated With High CD8 Counts in Immune Restored Patients on Long-Term ART. Front Immunol (2020) 11:1541. doi: 10.3389/fimmu.2020.01541 32793212 PMC7390854

[2] Gazzola L Tincati C Bellistri GM Monforte A Marchetti G. The Absence of CD4+ T Cell Count Recovery Despite Receipt of Virologically Suppressive Highly Active Antiretroviral Therapy: Clinical Risk, Immunological Gaps, and Therapeutic Options. Clin Infect Dis (2009) 48(3):328–37. doi: 10.1086/595851 19123868

[3] Corbeau P Reynes J. Immune Reconstitution Under Antiretroviral Therapy: The New Challenge in HIV-1 Infection. Blood (2011) 117(21):5582–90. doi: 10.1182/blood-2010-12-322453 21403129

[4] Yang X Su B Zhang X Liu Y Wu H Zhang T. Incomplete Immune Reconstitution in HIV/AIDS Patients on Antiretroviral Therapy: Challenges of Immunological Non-Responders. J Leukoc Biol (2020) 107(4):597–612. doi: 10.1002/JLB.4MR1019-189R 31965635 PMC7187275

[5] Kong Y Tian Y Hao Y Chong X Xiao J Yang D. Two Types of Poor Immunological Responder Showing Distinct Responses to Long-Term HAART. Int J Infect Dis (2019) 86:178–87. doi: 10.1016/j.ijid.2019.07.037 31398453

[6] Paiardini M Muller-Trutwin M. HIV-Associated Chronic Immune Activation. Immunol Rev (2013) 254(1):78–101. doi: 10.1111/imr.12079 23772616 PMC3729961

[7] Vidya Vijayan KK Karthigeyan KP Tripathi SP Hanna LE. Pathophysiology of CD4+ T-Cell Depletion in HIV-1 and HIV-2 Infections. Front Immunol (2017) 8:580. doi: 10.3389/fimmu.2017.00580 28588579 PMC5440548

[8] Lv T Cao W Li T. HIV-Related Immune Activation and Inflammation: Current Understanding and Strategies. J Immunol Res (2021) 2021:7316456. doi: 10.1155/2021/7316456 34631899 PMC8494587

[9] Naidoo KK Ndumnego OC Ismail N Dong KL Ndung’u T. Antigen Presenting Cells Contribute to Persistent Immune Activation Despite Antiretroviral Therapy Initiation During Hyperacute HIV-1 Infection. Front Immunol (2021) 12:738743. doi: 10.3389/fimmu.2021.738743 34630420 PMC8498034

[10] Zhang Y Jiang T Li A Li Z Hou J Gao M. Adjunct Therapy for CD4(+) T-Cell Recovery, Inflammation and Immune Activation in People Living With HIV: A Systematic Review and Meta-Analysis. Front Immunol (2021) 12:632119. doi: 10.3389/fimmu.2021.632119 33679779 PMC7925844

[11] Bandera A Masetti M Fabbiani M Biasin M Muscatello A Squillace N. The NLRP3 Inflammasome Is Upregulated in HIV-Infected Antiretroviral Therapy-Treated Individuals With Defective Immune Recovery. Front Immunol (2018) 9:214. doi: 10.3389/fimmu.2018.00214 29483915 PMC5816335

[12] Brandt D Hedrich CM. TCRalphabeta(+)CD3(+)CD4(-)CD8(-) (Double Negative) T Cells in Autoimmunity. Autoimmun Rev (2018) 17(4):422–30. doi: 10.1016/j.autrev.2018.02.001 29428806

[13] Neyt K GeurtsvanKessel CH Lambrecht BN. Double-Negative T Resident Memory Cells of the Lung React to Influenza Virus Infection *via* CD11c(hi) Dendritic Cells. Mucosal Immunol (2016) 9(4):999–1014. doi: 10.1038/mi.2015.91 26376363

[14] Merims S Li X Joe B Dokouhaki P Han M Childs RW. Anti-Leukemia Effect of *Ex Vivo* Expanded DNT Cells From AML Patients: A Potential Novel Autologous T-Cell Adoptive Immunotherapy. Leukemia (2011) 25(9):1415–22. doi: 10.1038/leu.2011.99 PMC4214360 21566657

[15] Hillhouse EE Lesage S. A Comprehensive Review of the Phenotype and Function of Antigen-Specific Immunoregulatory Double Negative T Cells. J Autoimmun (2013) 40:58–65. doi: 10.1016/j.jaut.2012.07.010 22910322

[16] Juvet SC Zhang L. Double Negative Regulatory T Cells in Transplantation and Autoimmunity: Recent Progress and Future Directions. J Mol Cell Biol (2012) 4(1):48–58. doi: 10.1093/jmcb/mjr043 22294241 PMC3269300

[17] Hillhouse EE Delisle JS Lesage S. Immunoregulatory CD4(-)CD8(-) T Cells as a Potential Therapeutic Tool for Transplantation, Autoimmunity, and Cancer. Front Immunol (2013) 4:6. doi: 10.3389/fimmu.2013.00006 23355840 PMC3553425

[18] Sundaravaradan V Saleem R Micci L Gasper MA Ortiz AM Else J. Multifunctional Double-Negative T Cells in Sooty Mangabeys Mediate T-Helper Functions Irrespective of SIV Infection. PloS Pathog (2013) 9(6):e1003441. doi: 10.1371/journal.ppat.1003441 23825945 PMC3694849

[19] Milush JM Mir KD Sundaravaradan V Gordon SN Engram J Cano CA. Lack of Clinical AIDS in SIV-Infected Sooty Mangabeys With Significant CD4+ T Cell Loss Is Associated With Double-Negative T Cells. J Clin Invest (2011) 121(3):1102–10. doi: 10.1172/JCI44876 PMC3049370 21317533

[20] Vinton C Klatt NR Harris LD Briant JA Sanders-Beer BE Herbert R. CD4-Like Immunological Function by CD4- T Cells in Multiple Natural Hosts of Simian Immunodeficiency Virus. J Virol (2011) 85(17):8702–8. doi: 10.1128/JVI.00332-11 PMC3165829 21715501

[21] Li Y Dong K Fan X Xie J Wang M Fu S. DNT Cell-Based Immunotherapy: Progress and Applications. J Cancer (2020) 11(13):3717–24. doi: 10.7150/jca.39717 PMC7171494 32328176

[22] Ford McIntyre MS Gao JF Li X Naeini BM Zhang L. Consequences of Double Negative Regulatory T Cell and Antigen Presenting Cell Interaction on Immune Response Suppression. Int Immunopharmacol (2011) 11(5):597–603. doi: 10.1016/j.intimp.2010.11.015 21109036

[23] Lu X Su B Xia H Zhang X Liu Z Ji Y. Low Double-Negative CD3(+)CD4(-)CD8(-) T Cells Are Associated With Incomplete Restoration of CD4(+) T Cells and Higher Immune Activation in HIV-1 Immunological Non-Responders. Front Immunol (2016) 7:579. doi: 10.3389/fimmu.2016.00579 28018346 PMC5145861

[24] Meziane O Salahuddin S Pham TNQ Farnos O Pagliuzza A Olivenstein R. HIV Infection and Persistence in Pulmonary Mucosal Double Negative T Cells *In Vivo* . J Virol (2020) 94(24):e01788–20. doi: 10.1128/JVI.01788-20 PMC7925170 32967958

[25] Petitjean G Chevalier MF Tibaoui F Didier C Manea ME Liovat AS. Level of Double Negative T Cells, Which Produce TGF-Beta and IL-10, Predicts CD8 T-Cell Activation in Primary HIV-1 Infection. AIDS (2012) 26(2):139–48. doi: 10.1097/QAD.0b013e32834e1484 22045342

[26] Zhang D Yang W Degauque N Tian Y Mikita A Zheng XX. New Differentiation Pathway for Double-Negative Regulatory T Cells That Regulates the Magnitude of Immune Responses. Blood (2007) 109(9):4071–9. doi: 10.1182/blood-2006-10-050625 PMC1874581 17197428

[27] Zhang ZX Ma Y Wang H Arp J Jiang J Huang X. Double-Negative T Cells, Activated by Xenoantigen, Lyse Autologous B and T Cells Using a Perforin/Granzyme-Dependent, Fas-Fas Ligand-Independent Pathway. J Immunol (2006) 177(10):6920–9. doi: 10.4049/jimmunol.177.10.6920 17082607

[28] Gao JF McIntyre MS Juvet SC Diao J Li X Vanama RB. Regulation of Antigen-Expressing Dendritic Cells by Double Negative Regulatory T Cells. Eur J Immunol (2011) 41(9):2699–708. doi: 10.1002/eji.201141428 21660936

[29] Ohta A Sitkovsky M. Extracellular Adenosine-Mediated Modulation of Regulatory T Cells. Front Immunol (2014) 5:304. doi: 10.3389/fimmu.2014.00304 25071765 PMC4091046

[30] Antonioli L Pacher P Vizi ES Hasko G. CD39 and CD73 in Immunity and Inflammation. Trends Mol Med (2013) 19(6):355–67. doi: 10.1016/j.molmed.2013.03.005 PMC3674206 23601906

[31] Meng F Guo Z Hu Y Mai W Zhang Z Zhang B. CD73-Derived Adenosine Controls Inflammation and Neurodegeneration by Modulating Dopamine Signalling. Brain (2019) 142(3):700–18. doi: 10.1093/brain/awy351 30689733

[32] Kong Y Jia B Zhao C Claxton DF Sharma A Annageldiyev C. Downregulation of CD73 Associates With T Cell Exhaustion in AML Patients. J Hematol Oncol (2019) 12(1):40. doi: 10.1186/s13045-019-0728-3 31014364 PMC6480867

[33] Schuler PJ Macatangay BJ Saze Z Jackson EK Riddler SA Buchanan WG. CD4(+)CD73(+) T Cells Are Associated With Lower T-Cell Activation and C Reactive Protein Levels and Are Depleted in HIV-1 Infection Regardless of Viral Suppression. AIDS (2013) 27(10):1545–55. doi: 10.1097/QAD.0b013e328360c7f3 PMC3939796 24005375

[34] Neo SY Yang Y Record J Ma R Chen X Chen Z. CD73 Immune Checkpoint Defines Regulatory NK Cells Within the Tumor Microenvironment. J Clin Invest (2020) 130(3):1185–98. doi: 10.1172/JCI128895 PMC7269592 31770109

[35] Beavis PA Stagg J Darcy PK Smyth MJ. CD73: A Potent Suppressor of Antitumor Immune Responses. Trends Immunol (2012) 33(5):231–7. doi: 10.1016/j.it.2012.02.009 22487321

[36] Chang W-X Huang H-H Huang L Shi J-J Jiao Y-M Zhang C. Skewed CD39/CD73/adenosine Pathway in B Cells Is Associated With Innate Immune Hyperactivation in Chronic HIV-1 Infection. Trans Med Commun (2019) 4(1):4. doi: 10.1186/s41231-019-0033-8

[37] Jenabian MA Seddiki N Yatim A Carriere M Hulin A Younas M. Regulatory T Cells Negatively Affect IL-2 Production of Effector T Cells Through CD39/adenosine Pathway in HIV Infection. PloS Pathog (2013) 9(4):e1003319. doi: 10.1371/journal.ppat.1003319 23658513 PMC3635970

[38] Nikolova M Carriere M Jenabian MA Limou S Younas M Kok A. CD39/adenosine Pathway Is Involved in AIDS Progression. PloS Pathog (2011) 7(7):e1002110. doi: 10.1371/journal.ppat.1002110 21750674 PMC3131268

[39] Lopez-Abente J Correa-Rocha R Pion M. Functional Mechanisms of Treg in the Context of HIV Infection and the Janus Face of Immune Suppression. Front Immunol (2016) 7:192. doi: 10.3389/fimmu.2016.00192 27242797 PMC4871867

[40] Deeks SG. HIV Infection, Inflammation, Immunosenescence, and Aging. Annu Rev Med (2011) 62:141–55. doi: 10.1146/annurev-med-042909-093756 PMC3759035 21090961

[41] Cohen J Torres C. HIV-Associated Cellular Senescence: A Contributor to Accelerated Aging. Ageing Res Rev (2017) 36:117–24. doi: 10.1016/j.arr.2016.12.004 PMC5584608 28017881

[42] Cotto B Natarajanseenivasan K Langford D. HIV-1 Infection Alters Energy Metabolism in the Brain: Contributions to HIV-Associated Neurocognitive Disorders. Prog Neurobiol (2019) 181:101616. doi: 10.1016/j.pneurobio.2019.101616 31108127 PMC6742565

[43] Serrao R Pinero C Velez J Coutinho D Maltez F Lino S. Non-AIDS-Related Comorbidities in People Living With HIV-1 Aged 50 Years and Older: The AGING POSITIVE Study. Int J Infect Dis (2019) 79:94–100. doi: 10.1016/j.ijid.2018.10.011 30529370

[44] Torti C Prosperi M Motta D Digiambenedetto S Maggiolo F Paraninfo G. Factors Influencing the Normalization of CD4+ T-Cell Count, Percentage and CD4+/CD8+ T-Cell Ratio in HIV-Infected Patients on Long-Term Suppressive Antiretroviral Therapy. Clin Microbiol Infect (2012) 18(5):449–58. doi: 10.1111/j.1469-0691.2011.03650.x 21919996

[45] Li CX Li YY He LP Kou J Bai JS Liu J. The Predictive Role of CD4(+) Cell Count and CD4/CD8 Ratio in Immune Reconstitution Outcome Among HIV/AIDS Patients Receiving Antiretroviral Therapy: An Eight-Year Observation in China. BMC Immunol (2019) 20(1):31. doi: 10.1186/s12865-019-0311-2 31455209 PMC6712592

[46] Hunt PW Cao HL Muzoora C Ssewanyana I Bennett J Emenyonu N. Impact of CD8+ T-Cell Activation on CD4+ T-Cell Recovery and Mortality in HIV-Infected Ugandans Initiating Antiretroviral Therapy. AIDS (2011) 25(17):2123–31. doi: 10.1097/QAD.0b013e32834c4ac1 PMC3480326 21881481

[47] Greenblatt R Bacchetti P Boylan R Kober K Springer G Anastos K. Genetic and Clinical Predictors of CD4 Lymphocyte Recovery During Suppressive Antiretroviral Therapy: Whole Exome Sequencing and Antiretroviral Therapy Response Phenotypes. PloS One (2019) 14(8):e0219201. doi: 10.1371/journal.pone.0219201 31415590 PMC6695188

[48] Rajasuriar R Gouillou M Spelman T Read T Hoy J Law M. Clinical Predictors of Immune Reconstitution Following Combination Antiretroviral Therapy in Patients From the Australian HIV Observational Database. PloS One (2011) 6(6):e20713. doi: 10.1371/journal.pone.0020713 21674057 PMC3107235

[49] Rb-Silva R Nobrega C Azevedo C Athayde E Canto-Gomes J Ferreira I. Thymic Function as a Predictor of Immune Recovery in Chronically HIV-Infected Patients Initiating Antiretroviral Therapy. Front Immunol (2019) 10:25. doi: 10.3389/fimmu.2019.00025 30804925 PMC6370619

[50] Sauter R Huang R Ledergerber B Battegay M Bernasconi E Cavassini M. CD4/CD8 Ratio and CD8 Counts Predict CD4 Response in HIV-1-Infected Drug Naive and in Patients on cART. Medicine (Baltimore) (2016) 95(42):e5094. doi: 10.1097/MD.0000000000005094 27759638 PMC5079322

[51] Guzman-Fulgencio M Berenguer J Jimenez-Sousa MA Micheloud D Garcia-Alvarez M Bellon JM. IL7RA Polymorphisms Predict the CD4+ Recovery in HIV Patients on cART. Eur J Clin Invest (2015) 45(11):1192–9. doi: 10.1111/eci.12539 26402121

[52] Guzman-Fulgencio M Berenguer J Micheloud D Fernandez-Rodriguez A Garcia-Alvarez M Jimenez-Sousa MA. European Mitochondrial Haplogroups Are Associated With CD4+ T Cell Recovery in HIV-Infected Patients on Combination Antiretroviral Therapy. J Antimicrob Chemother (2013) 68(10):2349–57. doi: 10.1093/jac/dkt206 23749950

[53] Kroeze S Rossouw TM Steel HC Wit FW Kityo CM Siwale M. Plasma Inflammatory Biomarkers Predict CD4+ T-Cell Recovery and Viral Rebound in HIV-1 Infected Africans on Suppressive Antiretroviral Therapy. J Infect Dis (2021) 224(4):673–8. doi: 10.1093/infdis/jiaa787 33373447

[54] Hernandez-Walias F Ruiz-de-Leon MJ Rosado-Sanchez I Vazquez E Leal M Moreno S. New Signatures of Poor CD4 Cell Recovery After Suppressive Antiretroviral Therapy in HIV-1-Infected Individuals: Involvement of miR-192, IL-6, Scd14 and miR-144. Sci Rep (2020) 10(1):2937. doi: 10.1038/s41598-020-60073-8 32076107 PMC7031287

[55] Chen X Liu X Duan S Tang R Zhou S Ye R. Plasma Inflammatory Biomarkers Associated With Advanced Liver Fibrosis in HIV–HCV-Coinfected Individuals. Int J Environ Res Public Health (2020) 17(24):9474. doi: 10.1097/QAD.0000000000002231 PMC7766690 33348839

[56] Watanabe D Uehira T Suzuki S Matsumoto E Ueji T Hirota K. Clinical Characteristics of HIV-1-Infected Patients With High Levels of Plasma Interferon-Gamma: A Multicenter Observational Study. BMC Infect Dis (2019) 19(1):11. doi: 10.1186/s12879-018-3643-2 30611204 PMC6321664

[57] Nguyen TP Shukla S Asaad R Freeman ML Lederman MM Harding CV. Responsiveness to IL-7 But Not to IFN-Alpha Is Diminished in CD4+ T Cells From Treated HIV Infected Patients Who Experience Poor CD4+ T-Cell Recovery. AIDS (2016) 30(13):2033–42. doi: 10.1097/QAD.0000000000001161 PMC5022566 27191978

[58] Fernandez S Tanaskovic S Helbig K Rajasuriar R Kramski M Murray JM. CD4+ T-Cell Deficiency in HIV Patients Responding to Antiretroviral Therapy Is Associated With Increased Expression of Interferon-Stimulated Genes in CD4+ T Cells. J Infect Dis (2011) 204(12):1927–35. doi: 10.1093/infdis/jir659 22006994

[59] Pino M Pereira Ribeiro S Pagliuzza A Ghneim K Khan A Ryan E. Increased Homeostatic Cytokines and Stability of HIV-Infected Memory CD4 T-Cells Identify Individuals With Suboptimal CD4 T-Cell Recovery on-ART. PloS Pathog (2021) 17(8):e1009825. doi: 10.1371/journal.ppat.1009825 34449812 PMC8397407

[60] Vaidya SA Korner C Sirignano MN Amero M Bazner S Rychert J. Tumor Necrosis Factor Alpha Is Associated With Viral Control and Early Disease Progression in Patients With HIV Type 1 Infection. J Infect Dis (2014) 210(7):1042–6. doi: 10.1093/infdis/jiu206 PMC4215080 24688071

[61] Reis EC Leal VNC da Silva LT Dos Reis MML Arganaraz ER Oshiro TM. Antagonistic Role of IL-1ss and NLRP3/IL-18 Genetics in Chronic HIV-1 Infection. Clin Immunol (2019) 209:108266. doi: 10.1016/j.clim.2019.108266 31669192

[62] Stiksrud B Lorvik KB Kvale D Mollnes TE Ueland PM Trseid M. Plasma IP-10 Is Increased in Immunological NonResponders and Associated With Activated Regulatory T Cells and Persisting Low CD4 Counts. J Acquir Immune Defic Syndr (2016) 73(2):138. doi: 10.1097/QAI.0000000000001080 27632144

[63] Erikstrup C Kronborg G Lohse N Rye OS Gerstoft J Ullum HJJ. T-Cell Dysfunction in HIV-1-Infected Patients With Impaired Recovery of CD4 Cells Despite Suppression of Viral Replication. J Acquir Immune Defic Syndr (2010) 53(3):303–10. doi: 10.1097/QAI.0b013e3181ca3f7c 20048679

[64] Prebensen C Ueland T Michelsen AE Lind A Pettersen FO Mollnes TE. High MIP-1β Levels in Plasma Predict Long-Term Immunological Nonresponse to Suppressive Antiretroviral Therapy in HIV Infection. (2015) 69(4):395–402. doi: 10.1097/QAI.0000000000000617 26115437

[65] Mussini C Lorenzini P Cozzi-Lepri A Lapadula G Marchetti G Nicastri E. CD4/CD8 Ratio Normalisation and Non-AIDS-Related Events in Individuals With HIV Who Achieve Viral Load Suppression With Antiretroviral Therapy: An Observational Cohort Study. Lancet HIV (2015) 2(3):e98–e106. doi: 10.1016/s2352-3018(15)00006-5 26424550

[66] Li B Zhang L Liu Y Xiao J Li C Fan L. A Novel Prediction Model to Evaluate the Probability of CD4/CD8 Ratio Restoration in HIV-Infected Individuals. AIDS (2022). doi: 10.1097/QAD.0000000000003167 35013083

[67] Serrano-Villar S Martínez-Sanz J Ron R Talavera-Rodríguez A Fernández-Felix BM Herrera S. Effects of First-Line Antiretroviral Therapy on the CD4/CD8 Ratio and CD8 Cell Counts in CoRIS: A Prospective Multicentre Cohort Study. Lancet HIV (2020) 7(8):e565–e73. doi: 10.1016/s2352-3018(20)30202-2 32763219

[68] Lu W Mehraj V Vyboh K Cao W Li T Routy JP. CD4:CD8 Ratio as a Frontier Marker for Clinical Outcome, Immune Dysfunction and Viral Reservoir Size in Virologically Suppressed HIV-Positive Patients. J Int AIDS Soc (2015) 18:20052. doi: 10.7448/IAS.18.1.20052 26130226 PMC4486418

[69] Roca-Bayerri C Robertson F Pyle A Hudson G Payne BAI. Mitochondrial DNA Damage and Brain Aging in Human Immunodeficiency Virus. Clin Infect Dis (2021) 73(2):e466–e73. doi: 10.1093/cid/ciaa984 PMC8282328 32722761

[70] Caby F Guiguet M Weiss L Winston A Miro JM Konopnicki D. CD4/CD8 Ratio and the Risk of Kaposi Sarcoma or Non-Hodgkin Lymphoma in the Context of Efficiently Treated Human Immunodeficiency Virus (HIV) Infection: A Collaborative Analysis of 20 European Cohort Studies. Clin Infect Dis (2021) 73(1):50–9. doi: 10.1093/cid/ciaa1137 34370842

[71] Mahapatra S Shearer WT Minard CG Mace E Paul M Orange JS. NK Cells in Treated HIV-Infected Children Display Altered Phenotype and Function. J Allergy Clin Immunol (2019) 144(1):294–303.e13. doi: 10.1016/j.jaci.2018.11.052 30735686 PMC8844948

[72] Serrano-Villar S Perez-Elias MJ Dronda F Casado JL Moreno A Royuela A. Increased Risk of Serious Non-AIDS-Related Events in HIV-Infected Subjects on Antiretroviral Therapy Associated With a Low CD4/CD8 Ratio. PloS One (2014) 9(1):e85798. doi: 10.1371/journal.pone.0085798 24497929 PMC3907380

[73] Kim ES Ackermann C Toth I Dierks P Eberhard JM Wroblewski R. Down-Regulation of CD73 on B Cells of Patients With Viremic HIV Correlates With B Cell Activation and Disease Progression. J Leukoc Biol (2017) 101(5):1263–71. doi: 10.1189/jlb.5A0816-346R 28193736

[74] Toth I Le AQ Hartjen P Thomssen A Matzat V Lehmann C. Decreased Frequency of CD73+CD8+ T Cells of HIV-Infected Patients Correlates With Immune Activation and T Cell Exhaustion. J Leukoc Biol (2013) 94(4):551–61. doi: 10.1189/jlb.0113018 23709688

[75] Carriere M Lacabaratz C Kok A Benne C Jenabian MA Casartelli N. HIV “Elite Controllers” Are Characterized by a High Frequency of Memory CD8+ CD73+ T Cells Involved in the Antigen-Specific CD8+ T-Cell Response. J Infect Dis (2014) 209(9):1321–30. doi: 10.1093/infdis/jit643 24357632

[76] Schneider E Winzer R Rissiek A Ricklefs I Meyer-Schwesinger C Ricklefs FL. CD73-Mediated Adenosine Production by CD8 T Cell-Derived Extracellular Vesicles Constitutes an Intrinsic Mechanism of Immune Suppression. Nat Commun (2021) 12(1):5911. doi: 10.1038/s41467-021-26134-w 34625545 PMC8501027

[77] Lehto MT Sharom FJ. Release of the Glycosylphosphatidylinositol-Anchored Enzyme Ecto-5’-Nucleotidase by Phospholipase C: Catalytic Activation and Modulation by the Lipid Bilayer. Biochem J (1998) 332(Pt 1):101. doi: 10.1042/bj3320101 9576857 PMC1219457

[78] Schneider E Rissiek A Winzer R Puig B Rissiek B Haag F. Generation and Function of Non-Cell-Bound CD73 in Inflammation. Front Immunol (2019) 10:1729. doi: 10.3389/fimmu.2019.01729 31404305 PMC6676417

[79] Wang M Jia J Cui Y Peng Y Jiang Y. CD73-Positive Extracellular Vesicles Promote Glioblastoma Immunosuppression by Inhibiting T-Cell Clonal Expansion. Cell Death Dis (2021) 12(11):1065. doi: 10.1038/s41419-021-04359-3 34753903 PMC8578373

[80] Botta Gordon-Smith S Ursu S Eaton S Moncrieffe H Wedderburn LR. Correlation of Low CD73 Expression on Synovial Lymphocytes With Reduced Adenosine Generation and Higher Disease Severity in Juvenile Idiopathic Arthritis. Arthritis Rheumatol (2015) 67(2):545–54. doi: 10.1002/art.38959 PMC5024010 25418634

[81] Clayton A Al-Taei S Webber J Mason MD Tabi Z. Cancer Exosomes Express CD39 and CD73, Which Suppress T Cells Through Adenosine Production. J Immunol (2011) 187(2):676–83. doi: 10.4049/jimmunol.1003884 21677139

[82] Hixson EA Borker PV Jackson EK Macatangay BJ. The Adenosine Pathway and Human Immunodeficiency Virus-Associated Inflammation. Open Forum Infect Dis (2021) 8(9):ofab396. doi: 10.1093/ofid/ofab396 34557556 PMC8454523

[83] Hasko G Cronstein B. Regulation of Inflammation by Adenosine. Front Immunol (2013) 4:85. doi: 10.3389/fimmu.2013.00085 23580000 PMC3619132

[84] Lofgren L Pehrsson S Hagglund G Tjellstrom H Nylander S. Accurate Measurement of Endogenous Adenosine in Human Blood. PloS One (2018) 13(10):e0205707. doi: 10.1371/journal.pone.0205707 30359421 PMC6201894

[85] Goding JW Grobben B Slegers H. Physiological and Pathophysiological Functions of the Ecto-Nucleotide Pyrophosphatase/Phosphodiesterase Family. Biochim Biophys Acta (BBA) - Mol Basis Dis (2003) 1638(1):1–19. doi: 10.1016/s0925-4439(03)00058-9 12757929

[86] Alcedo KP Bowser JL Snider NT. The Elegant Complexity of Mammalian Ecto-5′-Nucleotidase (CD73). Trends Cell Biol (2021) 31(10):829–42. doi: 10.1016/j.tcb.2021.05.008 PMC8448938 34116887

