# Supplementary material for: Decreased CD73+ Double-Negative T Cells and Elevated Level of Soluble CD73 Correlated With and Predicted Poor Immune Reconstitution in HIV-Infected Patients After Antiretroviral Therapy
Source: Front Immunol. 2022 Apr 4;13:869286. doi: 10.3389/fimmu.2022.869286 (PMC9013806; doi:10.3389/fimmu.2022.869286)
Supplement: Supplementary file 1 [file DataSheet_1.pdf]

Supplementary Material

Supplementary Figures

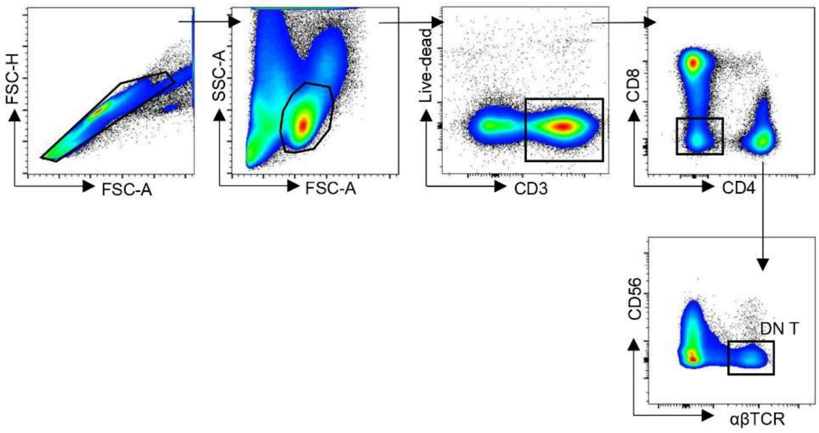

Fig. S1 Identification of αβ DNT cells

Representative gating strategy for flow cytometry analysis of αβ DNT cells in PLWH.

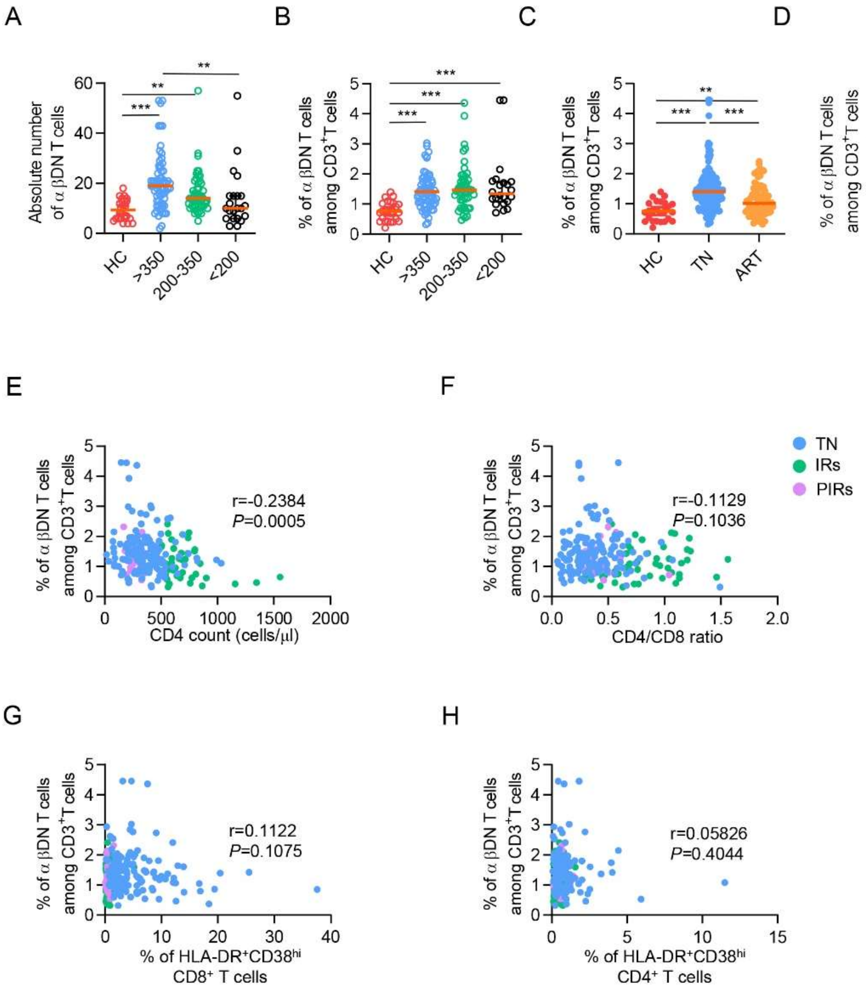

Fig. S2 Detection of absolute number and frequencies of DNT cells in HCs, TNs, IRs and PIRs

(A) Scatter plots compared the absolute number of DNT cells from HCs and TNs (n=22-66 each group). *P* values were obtained by the Kruskal-Wallis test followed by Dunn's multiple comparisons test. (B-D) Frequencies of DNT cells among $CD3^{+}$ T cells from HCs, TNs, IRs and PIRs (n=22-139 each group). *P* values were obtained by Kruskal-Wallis test followed by Dunn's multiple comparisons test and unpaired t-test. (E-H) Correlation between frequency of DNT cells among $CD3^{+}$T cells with $CD4^{+}$ T cell count (E), CD4/CD8 ratio (F), HLA-$DR^{+}CD38^{hi}$ $CD8^{+}$ T cells (G), and HLA-$DR^{+}CD38^{hi}$ $CD4^{+}$ T cells (H). Spearman's non-parametric test was used to test for correlations. $^{**}P<0.01$, $^{***}P<0.001$.

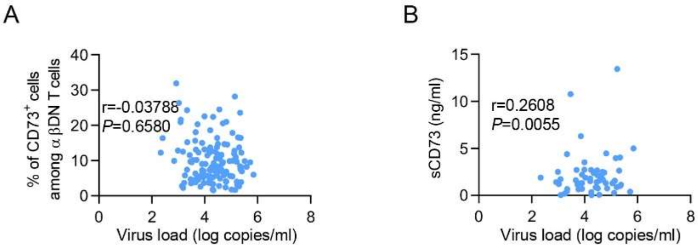

Fig. S3 Correlation of virus load with CD73$^+$ DNT frequency and sCD73 level

Correlation of virus load with the frequency of CD73$^+$ DNT cells (A, n=139) and the concentration of sCD73 in baseline (B, n=58). Spearman's non-parametric test was used to test for correlations.

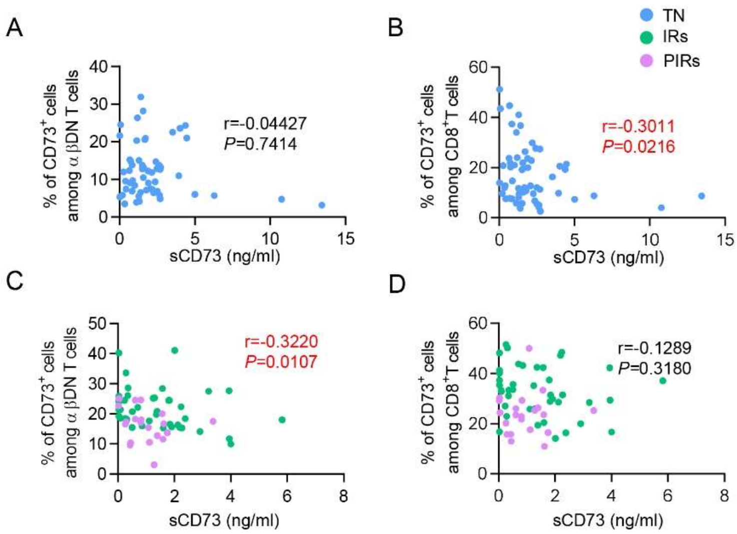

Fig. S4 Correlation of sCD73 level with frequencies of CD73$^+$ DNT cells and CD73$^+$ CD8$^+$ T cells

(A-B) Correlation of sCD73 level with the frequency of CD73$^+$ DNT cells (A) and CD73$^+$ CD8$^+$ T cells (B) in TNs (n=58). (C-D) Correlation of sCD73 level with the frequency of CD73$^+$ DNT cells (C) and CD73$^+$ CD8$^+$ T cells (D) in IRs and PIRs (n=41 for IRs, n=21 for PIRs). Spearman's non-parametric test was used to test for correlations.

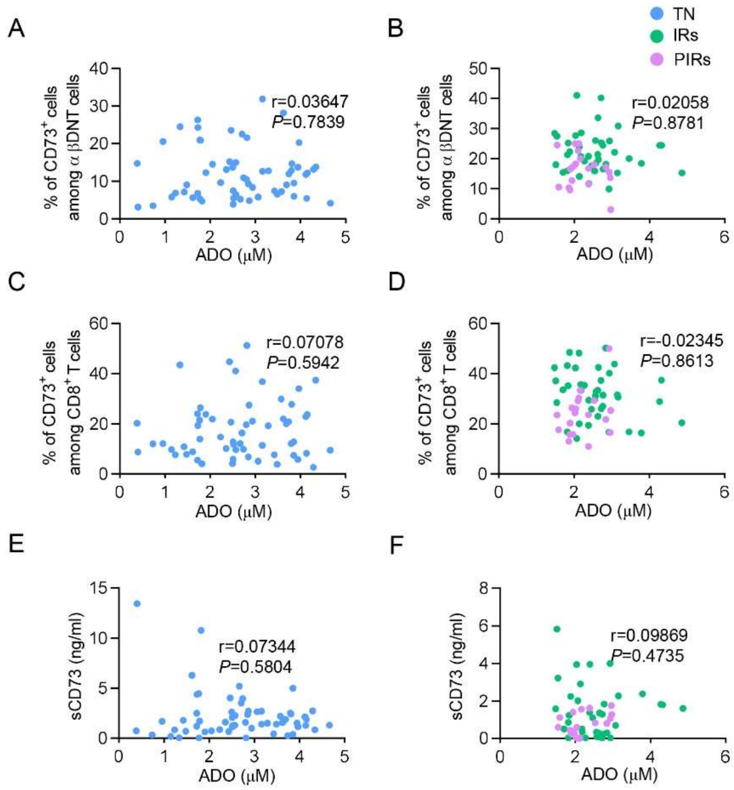

Fig. S5 Correlation of ADO level with frequencies of CD73^{+} DNT cells, CD73^{+} CD8^{+} T cells, and sCD73

(A-B) Correlation of ADO level with the frequency of CD73^{+} DNT cells in TNs (n=59) and in IRs and PIRs (n=39 for IRs, n=19 for PIRs). (C-D) Correlation of ADO level with the frequency of CD73^{+} CD8^{+} T cells in TNs (n=59) and in IRs and PIRs (n=39 for IRs, n=19 for PIRs). (E-F) Correlation of ADO level with the sCD73 in TNs (n=59) and in IRs and PIRs (n=39 for IRs, n=19 for PIRs). Spearman's non-parametric test and Pearson correlation test was used to test for correlations.

Supplementary Material

Supplementary Tables

Supplementary Table 1 Demographic and clinical characteristics of participants in the retrospective cohort study

Characteristics	All patients (n=171)
Sex (M/F)	169/2
Age (mean, years)	36±10
Baseline $CD4^+$ T cell count (cells/mm^3), median (IQR)	171(43-262)
Baseline $CD8^+$ T cell count (cells/mm^3), median (IQR)	834(620-1101)
Baseline CD4/CD8 ratio at baseline, median (IQR)	0.18(0.06-0.30)
Baseline HIV RNA viral load (copies/mL), median (IQR)	67747(26842-180500)

Abbreviations: PLWH, people living with HIV; M, male; F, female.

Supplementary Table 2 Incidence of poor immune reconstitution in groups of high and low sCD73 at different time-points post ART

	High-sCD73 (n=70)	Low-sCD73 (n=101)	*P*
1-year[a]	48/69 (69.6%)	53/101 (52.5%)	**0.026**
2-year[b]	41/70 (58.6%)	43/100(43.0%)	**0.046**
3-year[c]	34/68 (50.0%)	30/101 (29.7%)	**0.008**
4-year[d]	35/69 (50.7%)	28/101 (27.7%)	**0.002**
5-year[e]	37/60 (61.7%)	24/93 (25.8%)	**<0.0001**

[a]One patient of high-sCD73 lost the result of $CD4^+$ T cell count.
[b]One patient of low-sCD73 lost the result of $CD4^+$ T cell count.
[c]Two patients of high-sCD73 lost the results of $CD4^+$ T cell count.
[d]One patient of high-sCD73 lost the result of $CD4^+$ T cell count.
[e]Ten patients of high-sCD73 lost the results of $CD4^+$ T cell count and eight patients of low-sCD73 lost the results of $CD4^+$ T cell count.
P values were obtained by Chi-square test. Bold numbers represent $P<0.05$
Abbreviations: ART, antiretroviral therapy; sCD73, soluble cluster of differentiation 73.

Supplementary table 3 Univariable and multivariable proportional hazards models of soluble plasma factors

	univariable		multivariable	
Baseline factors	HR (95%CI)	*P*	HR (95%CI)	*P*
**IL-1β**	**1.578 (1.084-2.296)**	**0.017**		
IL-2	1.045 (0.707-1.543)	0.825		
IL-7	1.702 (0.986-2.938)	0.056		
IL-15	1.053 (0.719-1.542)	0.792		
**IL-18**	**1.736 (1.145-2.634)**	**0.009**		
IFN-α	1.395 (0.959-2.029)	0.081		
**IFN-γ**	**1.750 (1.164-2.632)**	**0.007**		
IP-10	1.320 (0.873-1.995)	0.188		
MIP-1β	1.238 (0.840-1.824)	0.281		
RANTES	0.925 (0.581-1.474)	0.743		
**sCD73**	**2.069 (1.397-3.063)**	**<0.0001**	**2.057 (1.356-3.121)**	**0.001**
**TNF-α**	**2.176 (1.433-3.304)**	**<0.0001**	**1.913 (1.241-2.950)**	**0.003**

Multivariate analysis is adjusted for all variables with $P<0.05$ in univariate analysis. Bold numbers represent $P<0.05$
Abbreviations: HR=hazard ratio
